# Supplementary material for: Tracking kdr alleles associated with pyrethroid resistance in Aedes albopictus across Italy: a nationwide genotypic dataset by MosqIRIT network
Source: GigaByte. 2026 Jul 2;2026:gigabyte185. doi: 10.46471/gigabyte.185 (PMC13359220; doi:10.46471/gigabyte.185)
Supplement: Peer review history

# Reviewer report: Review MS


Reviewer name and names of any other individual's who aided in reviewer Zijun XiongDo you understand and agree to our policy of having open and named reviews, and having your review included with the published papers. (If no, please inform the editor that you cannot review this manuscript.)YesIs the language of sufficient quality?YesPlease add additional comments on language quality to clarify if needed
Are all data available and do they match the descriptions in the paper? NoAdditional CommentsI would suggest the authors to also upload the raw sequencing data to GigaDB.Are the data and metadata consistent with relevant minimum information or reporting standards? See GigaDB checklists for examples <a href="http://gigadb.org/site/guide" target="_blank">http://gigadb.org/site/guide</a>YesAdditional CommentsIs the data acquisition clear, complete and methodologically sound?YesAdditional CommentsIs there sufficient detail in the methods and data-processing steps to allow reproduction?YesAdditional CommentsIs there sufficient data validation and statistical analyses of data quality? YesAdditional CommentsIs the validation suitable for this type of data?YesAdditional CommentsIs there sufficient information for others to reuse this dataset or integrate it with other data?NoAdditional CommentsThe authors should provide detailed guideline on how to access and use itAny Additional Overall Comments to the AuthorThe manuscript present a nationwide genotypic dataset generated by the MosqIRIT Network, comprising a carefully curated and georeferenced resource on the frequencies of the two primary target-site mutations associated with pyrethroid resistance in Aedes albopictus across Italy. The manuscript is very well written, and the data are solid, clearly presented, and thoughtfully analyzed. I particularly appreciate the authors’ efforts to integrate environmental and demographic variables. Surprisingly (to me), I actually do not have any major issues with the manuscript in its current shape! The authors could more clearly highlight the annotated genotypic resource and provide clearer guidance on how to access and use it, so that many other labs will be able to benefit from the data and cite it.RecommendationAccept


# Reviewer report: Review MS


Reviewer name and names of any other individual's who aided in reviewer Yan PengDo you understand and agree to our policy of having open and named reviews, and having your review included with the published papers. (If no, please inform the editor that you cannot review this manuscript.)YesIs the language of sufficient quality?YesPlease add additional comments on language quality to clarify if needed
Are all data available and do they match the descriptions in the paper? YesAdditional CommentsAre the data and metadata consistent with relevant minimum information or reporting standards? See GigaDB checklists for examples <a href="http://gigadb.org/site/guide" target="_blank">http://gigadb.org/site/guide</a>YesAdditional CommentsIs the data acquisition clear, complete and methodologically sound?YesAdditional CommentsIs there sufficient detail in the methods and data-processing steps to allow reproduction?YesAdditional CommentsIs there sufficient data validation and statistical analyses of data quality? YesAdditional CommentsIs the validation suitable for this type of data?YesAdditional CommentsIs there sufficient information for others to reuse this dataset or integrate it with other data?YesAdditional CommentsThe authors conducted population sampling across 102 of the 107 provinces in Italy. Genotyping was performed on 3,517 individuals using allele-specific PCR assays. This study is of great significance for pest control and monitoring, and the dataset provides a valuable reference for mosquito surveillance programs. However, several detailed issues in the manuscript need to be revised prior to publication. The authors have provided substantial information, yet Figure 1 only displays differences in frequency. The sample size for each data point should also be presented, as the large variation in total sample numbers may lead to biased estimates of the locus frequencies. Additionally, it would be preferable to present the data stratified by year and month. Furthermore, it is necessary to clarify whether there is a linkage relationship between these two mutations. Were both variants detected simultaneously in a single individual during variant genotyping? If so, the results could be integrated for analysis. Given such a large-scale sampling effort, it would be highly beneficial to briefly infer the temporal trends by incorporating environmental data and key events. This would significantly enhance the analytical depth of the dataset. Naturally, this section can be further elaborated in the Discussion.Any Additional Overall Comments to the AuthorRecommendationMinor Revision


# Editor report: Accept


Editor’s AssessmentThe manuscript is ready for formal acceptance.Editor’s AssessmentThe manuscript is ready for formal acceptance.
